# Understanding the Hydrothermal Formation of NaNbO_3_: Its Full Reaction Scheme and Kinetics

**DOI:** 10.1021/acs.inorgchem.0c02763

**Published:** 2021-03-23

**Authors:** Susanne Linn Skjærvø, Gary K. Ong, Ola Gjønnes Grendal, Kristin Høydalsvik Wells, Wouter van Beek, Koji Ohara, Delia J. Milliron, Satoshi Tominaka, Tor Grande, Mari-Ann Einarsrud

**Affiliations:** † Department of Materials Science and Engineering, NTNU Norwegian University of Science and Technology, 7491 Trondheim, Norway; ‡ McKetta Department of Chemical Engineering, The University of Texas at Austin, Austin, Texas 78712, United States; § Swiss-Norwegian Beamlines at the European Synchrotron Radiation Facility, 71 Avenue des Martyrs, 38043 Grenoble Cedex 9, France; ∥ Diffraction and Scattering Division, Center for Synchrotron Radiation Research, Japan Synchrotron Radiation Research Institute, 1-1-1 Kouto, Sayo-cho, Sayo-gun, Hyogo 679-5198, Japan; ⊥ International Center for Materials Nanoarchitectonics (WPI-MANA), National Institute for Materials Science (NIMS), 1-1 Namiki, Tsukuba, Ibaraki 305-0044, Japan

## Abstract

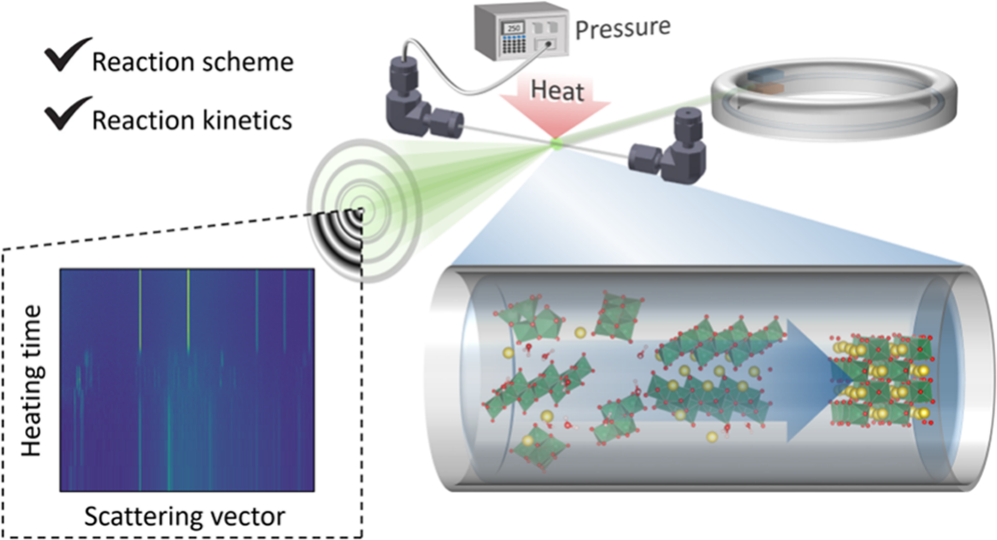

Sodium niobate (NaNbO_3_) attracts attention for its great potential in a variety of applications, for instance, due to its unique optical properties. Still, optimization of its synthetic procedures is hard due to the lack of understanding of the formation mechanism under hydrothermal conditions. Through *in situ* X-ray diffraction, hydrothermal synthesis of NaNbO_3_ was observed in real time, enabling the investigation of the reaction kinetics and mechanisms with respect to temperature and NaOH concentration and the resulting effect on the product crystallite size and structure. Several intermediate phases were observed, and the relationship between them, depending on temperature, time, and NaOH concentration, was established. The reaction mechanism involved a gradual change of the local structure of the solid Nb_2_O_5_ precursor upon suspending it in NaOH solutions. Heating gave a full transformation of the precursor to HNa_7_Nb_6_O_19_·15H_2_O, which destabilized before new polyoxoniobates appeared, whose structure depended on the NaOH concentration. Following these polyoxoniobates, Na_2_Nb_2_O_6_·H_2_O formed, which dehydrated at temperatures ≥285 °C, before converting to the final phase, NaNbO_3_. The total reaction rate increased with decreasing NaOH concentration and increasing temperature. Two distinctly different growth regimes for NaNbO_3_ were observed, depending on the observed phase evolution, for temperatures below and above ≈285 °C. Below this temperature, the growth of NaNbO_3_ was independent of the reaction temperature and the NaOH concentration, while for temperatures ≥285 °C, the temperature-dependent crystallite size showed the characteristics of a typical dissolution–precipitation mechanism.

## Abstract 2

To understand the pathway from T-Nb_2_O_5_ to hydrothermally produced NaNbO_3_, the reaction was studied *in situ* with powder X-ray diffraction, small-angle scattering, and total scattering with pair-distribution function analysis. The full reaction scheme and kinetics were revealed, showing two different temperature-dependent growth mechanisms.

## Introduction

Hydrothermal synthesis is a low-temperature environmentally friendly route to a variety of functional oxides reducing challenges with evaporation, agglomeration, and coarsening, which often takes place at higher temperatures.^1−6^ Still, the development of the method has been mostly achieved through a trial-and-error approach as the conventional autoclave design, not easily penetrable by X-rays, makes it inherently challenging to study the synthesis in real time. Thus, the nature of the reactions taking place inside the reaction vessel is not completely understood.

NaNbO_3_ has gained attention due to its many potential applications in high-density optical storage, enhancing nonlinear optical properties, as hologram recording materials, *etc*.,^7,8^ It is also an end-member of the K*_x_*Na_1–*x*_NbO_3_ solid solution, a promising lead-free replacement for lead zirconate titanate (PZT).^9,10^ Moreover, NaNbO_3_ nanowires formed by hydrothermal synthesis and subsequent calcination have proven useful in lead-free piezoelectric nanogenerator applications.^11^*Ex situ* studies of the hydrothermal synthesis of NaNbO_3_^12−17^ have shown that the reaction starts with the transformation of the T-Nb_2_O_5_ (orthorhombic structure) precursor into sodium hexaniobate (HNa_7_Nb_6_O_19_·15H_2_O), with the main building block consisting of the Lindqvist ion, [Nb_6_O_19_]^8–^.^18^ The sodium hexaniobate then transforms into Na_2_Nb_2_O_6_·H_2_O, which in turn transforms into perovskite NaNbO_3_, displaying a wide range of morphologies including cubes^19−21^ and various agglomerated structures.^17,22,23^ The crystal structures of these phases are significantly different from each other, as seen in Figure S1 in the Supporting Information, and it is not clear how the structures evolve from one phase to the next or how they affect the growth mechanism of NaNbO_3_. Some attempts have been made to understand these growth mechanisms by *ex situ* studies, including the effect of the precursor^19^ and some intermediate structures,^20^ but as recent *in situ* studies have shown the presence of several more intermediate phases than previously reported,^24,25^ the proposed growth mechanisms may not give a full depiction of the resulting effects on the NaNbO_3_ growth. More work is therefore needed to understand how these reaction schemes depend on temperature and mineralizer concentration and how the product is consequently affected.

Here, we present an *in situ* X-ray diffraction (XRD) study of hydrothermal synthesis of NaNbO_3_, shedding light on the entire reaction scheme for a wide range of synthesis temperatures and NaOH concentrations, commonly seen in the literature.^14,20−22,26^ We determine how the reaction scheme is affected by reaction temperature and NaOH concentration. Knowledge about the kinetics during formation of NaNbO_3_, which is affected by the reaction mechanism, is obtained and useful for the optimization of reaction rate and resulting crystallite size. Further, as most literature on hydrothermal synthesis of NaNbO_3_ presents data at temperatures below 250 °C, we investigate the reaction at higher temperatures. In combination, the acquired knowledge provides the ability to speed up the reaction while still being able to achieve the desired reaction product, valuable for production at industrial scales.

## Experimental Section

Orthorhombic T-Nb_2_O_5_ powder^27^ was synthesized by precipitation from (NH_4_)NbO(C_2_O_4_)_2_·5H_2_O (Sigma-Aldrich, 99.99%) dissolved in water by adding aqueous ammonia solution (25 wt %, Emsure) before drying and then calcining at 600 °C for 12 h, as described by Mokkelbost et al.^25,28^ Highly concentrated suspensions were made by mixing T-Nb_2_O_5_ powder with 9 or 12 M NaOH aqueous solutions, giving a Na/Nb ratio of 9.5 or 13.2. The suspensions were stored in PET bottles and injected with a plastic syringe into a custom-made *in situ* cell, making sure to fill the entire volume of the cell. The cell, which has been previously described,^25^ consisted of a sapphire capillary with inner and outer diameters of 0.8 and 1.15 mm, respectively, which was fixed to an adjustable aluminum frame by graphite ferrules and Swagelok fittings. A High Pressure Liquid Chromatography (HPLC) pump connected to the dead-ended cell provided a stable pressure. The mid 1/3 of the capillary’s length was heated by a hot-air blower, and the temperature was calibrated by refining the unit cell expansion of boron nitride.^29^ The blower was ramped up to reaction temperature while being directed away from the capillary and was remotely swung into position only after the desired pressure was achieved and data acquisition had been initiated, providing quasi-instant heating (see temperature profiles in Figure S2 in the Supporting Information). Temperatures in the range of 160–420 °C were studied, and the pressure was set to 250 bar.

*In situ* powder X-ray diffraction (PXRD) data were collected at the Swiss-Norwegian Beamlines (BM01) at the European Synchrotron Radiation Facility (ESRF) using a monochromatic beam with a wavelength of 0.6776 Å. The diffraction signal was detected by a Pilatus 2M detector^30^ with acquisition times of 0.1 or 5 s depending on the experiment. The as-recorded data were treated with the Pilatus@SNBL platform,^30^ and the refinements were performed using TOPAS (version 5) in launch mode using JEdit with macros for TOPAS.^31^ Batch refinements were made possible by launching TOPAS with Jupyter Lab/Notebook.^32^ The diffraction patterns were compared to structure files from the Inorganic Crystal Structure Database (ICSD), Crystallographic Open Database (COD), and International Centre for Diffraction Data (ICDD). Phases with adequate signal/noise ratio, which could not be fitted successfully to any known structures, were indexed by a grid search using McMaille^33^ on the 20 most intense diffraction lines. The choice of a proper unit cell was based on a high figure of merit, provided that all of the reflections were identified. The least symmetric space group was then chosen to avoid extinction of any reflections.

The instrumental resolution function, wavelength, and detector distance were found and calibrated by refining an NIST 660a LaB_6_ standard. The diffraction patterns of the product phases were summed (25–60 s total acquisition time) to enhance statistics, and the default approach was a Rietveld refinement, refining the unit cell parameters, the Gaussian and Lorentzian isotropic size parameters, isotropic temperature factors, scale factor, and Chebychev background parameters. The Lorentzian and Gaussian isotropic size parameters were used to extract the integral breadth to give volume-weighted mean crystallite size. The intermediate phases HNa_7_Nb_6_O_19_·15H_2_O and Na_2_Nb_2_O_6_·H_2_O were refined with the space groups *Pmnn*(34) and *C*2/*c*,^35^ and the NaNbO_3_ product was refined with the space groups *Pbcm*(36) or *Pnma*.^37^ Atomic positions in all of the structures were fixed to literature values. For the time-resolved Rietveld refinements, the same approach as described above was used, but with additionally fixing the isotropic temperature factors. The phase fraction evolution of NaNbO_3_ was assumed to correspond to the normalized time-resolved scale factor. Structures were visualized with VESTA.^38^ Information about the kinetics of the NaNbO_3_ growth mechanism was extracted by fitting the refined phase fraction over time to the Johnson–Mehl–Avrami (JMA) equation.^39,40^

Simultaneous *in situ* small-angle X-ray scattering (SAXS) and PXRD were performed at beamline 7.3.3 at the Advanced Light Source in Berkeley, California, using monochromatic X-rays of 1.2398 Å. The PXRD and SAXS signal were acquired with a Pilatus 300K-W detector and a Pilatus 2M detector, respectively. The instrument geometry configuration was calibrated using a silver behenate (CH_3_(CH_2_)_20_COOAg) standard. The two-dimensional data were reduced using the Nika Igor Pro analysis software package.^100^ The data were plotted and analyzed in Jupyter Lab^32^ with the Scipy tool packages Numpy, Matplotlib, and Pandas.^41^ The suspensions made for this purpose had 50 wt % of T-Nb_2_O_5_ compared to the *in situ* PXRD experiments performed at the ESRF (as described above) to enhance the X-ray transmission. The same *in situ* cell as described above was used, but with a splash protection cage of aluminum bars and Kapton films around it.

Total scattering measurements on unheated suspensions of T-Nb_2_O_5_ powder in NaOH solutions at ambient pressure were performed at beamline BL08W at SPring-8/JASRI in Hyogo, Japan, using an a-Si flat panel area detector.^42^ The wavelength (0.1077 Å) and instrumental parameters were calibrated with an NIST 660a CeO_2_ standard. Similar suspensions to those for the *in situ* PXRD experiments, with 9 and 12 M NaOH solutions, fresh and aged for 1, 10, and 24 h were injected into 0.5 mm Kapton capillaries. The transmission signal was detected with 1 s acquisition time, collecting a total of 10 images per sample. The data were background-subtracted and converted to reduced structure functions, *F*(*Q*), and then Fourier-transformed to pair-distribution functions (PDF), *G*(*r*),^43^ using xPDFsuite^44^ and analyzed using the Diffpy-CMI software using a *Q*_max_ of 16.5 Å^–1^ and a *Q*_min_ of 1.2 Å^–1^.^45^

## Results and Discussion

All of the experiments were performed using the same T-Nb_2_O_5_ solid precursor suspended in 9 and 12 M NaOH aqueous solutions. All of the suspensions were hydrothermally treated at 250 bar in the temperature range 160–285 °C. An additional reaction with 9 M NaOH was monitored under supercritical conditions (250 bar, 420 °C). The effects of NaOH concentration and reaction temperature on the phase evolution, crystallite size, unit cell volume, and reaction kinetics are discerned in the following sections. Note that the datasets for 9 and 12 M NaOH at 215 °C and 9 M NaOH at 420 °C have been published previously.^25^

### Effects of Reaction Conditions on the Phase Evolution

**Figure 1:**
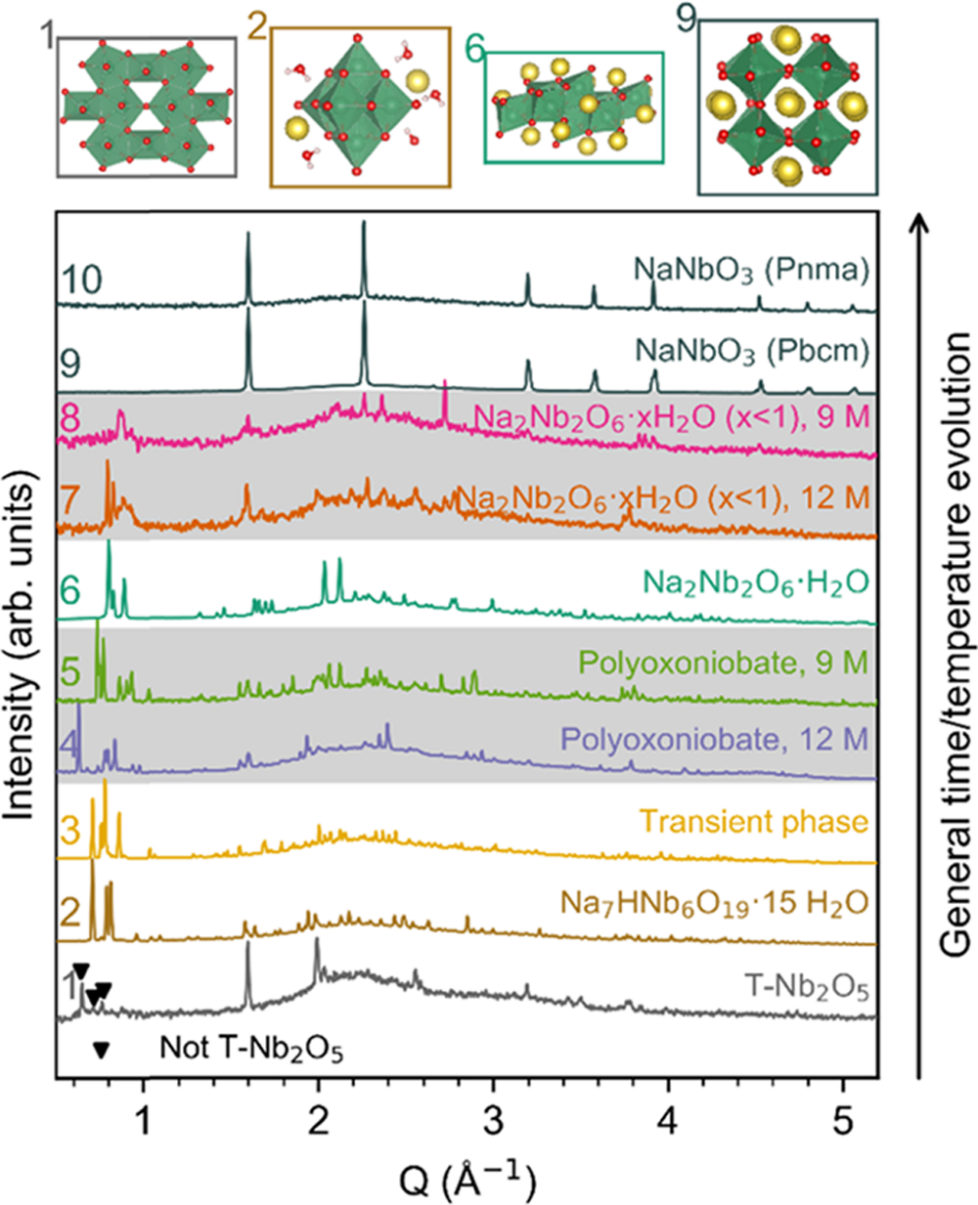
X-ray diffraction patterns for all appearing phases, with the main structural element for the previously known phases^27,34−37^ shown on the top. Gray areas indicate phases appearing at the same step in the reaction, but for different NaOH concentrations. Each diffraction pattern was taken from the slowest proceeding reaction (*i*.*e*., lowest temperature) and lowest possible NaOH concentration, where the phase was present, to optimize statistics.

Figure 1 shows the diffraction patterns of all of the phases observed during the performed experiments, numbered 1–10, for different heating time and/or temperature, leading to the formation of NaNbO_3_ at 160–420 °C. The main structural elements of the previously known phases^27,34−36^ are shown on the top of the figure, and their full structures are shown in Figure S1 in the Supporting Information. The patterns in the gray areas are pH variants at equivalent times in the reaction.

The diffraction lines of T-Nb_2_O_5_ (no. 1 in Figure 1) are seen in the diffraction pattern of the unheated precursor suspension with the addition of three diffraction lines at very low *Q* (0.65, 0.71, 0.76 Å^–1^). The presence of these lines seemed to be dependent on the time since the suspensions were made, which will be investigated, along with their origin, in later paragraphs. All of the diffraction patterns except for the product, NaNbO_3_ (nos. 9 and 10 in Figure 1), had diffraction lines at similarly low *Q*-values, demonstrating the large unit cells of phases present.

**Figure 2:**
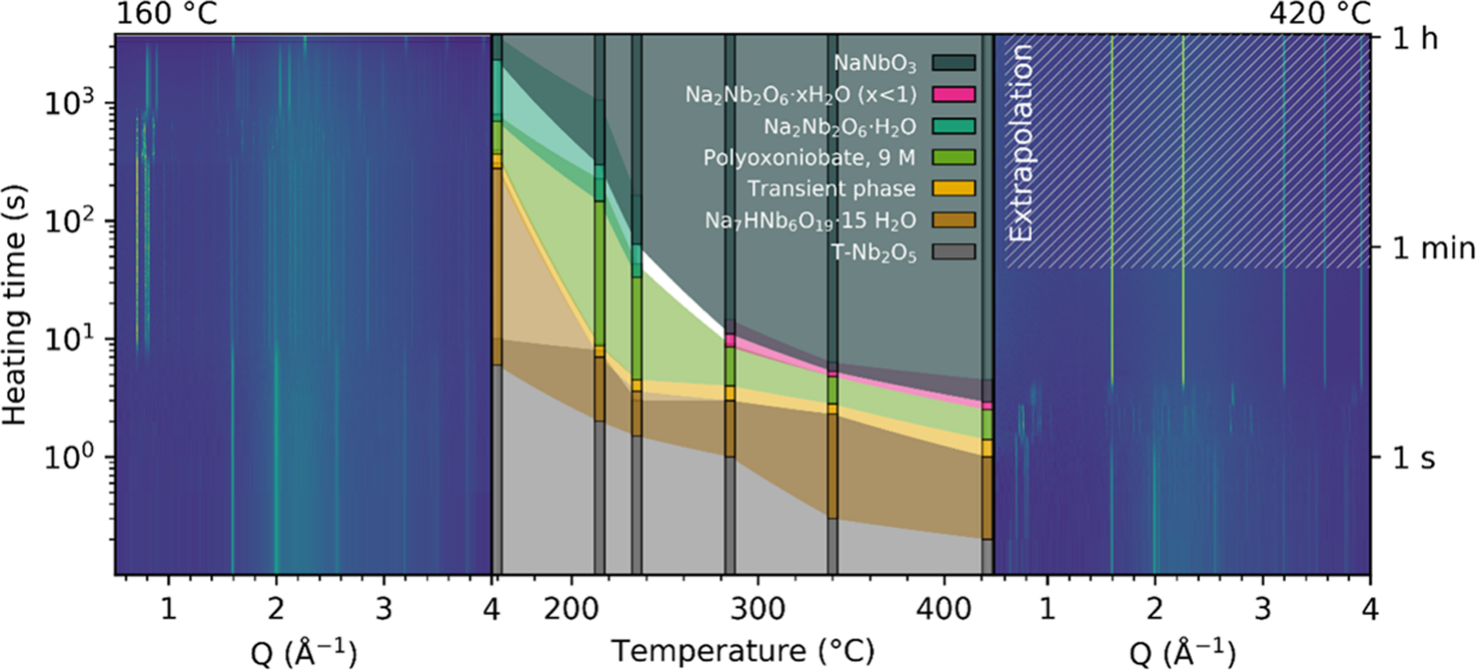
Qualitative time-resolved phase evolution during hydrothermal synthesis of NaNbO_3_ in 9 M NaOH aqueous solutions as a function of reaction temperature. Vertical bars represent real measured data, while the colored areas between them are logarithmic interpolations. The areas are transparent to make overlapping phases visible. Complete contour plots at the temperature limits are shown at each side. Contour plot for 9 M NaOH at 420 °C has been adapted with permission from ref (25) (Copyright © 2018 American Chemical Society).

In agreement with our previously reported paper,^25^ the T-Nb_2_O_5_ precursor (no. 1 in Figure 1) is transformed into HNa_7_Nb_6_O_19_·15H_2_O (no. 2 in Figure 1), before several intermediate phases form (nos. 3–5 in Figure 1), ending with the formation of Na_2_Nb_2_O_6_·H_2_O (no. 6 in Figure 1) and NaNbO_3_ (nos. 9 and 10 in Figure 1). In this work, the temperature dependency of this phase evolution has been identified and is presented in Figure 2, where the data for an expanded temperature region (160–420 °C) for suspensions with 9 M NaOH are shown (the equivalent data for 12 M NaOH at 160–285 °C are presented in Figure S3 in the Supporting Information). Contour plots for the end temperatures are shown at each side. The colored region in the middle section of the figure shows bar plots representing the recorded phase evolution at certain temperatures, with logarithmic interpolations between them. The reaction scheme appearing in the 9 and 12 M NaOH solutions are quite similar at similar temperatures, and the reaction rate increases in a comparable manner for increasing temperature for both concentrations. All of the reactions finished with a full conversion to NaNbO_3_, except in 12 M NaOH at 160 °C, where the experiment was prematurely stopped after an almost complete conversion to NaNbO_3_ (contour plots of both experiments at 160 °C are also shown with a linear *y*-axis in Figure S4 in the Supporting Information). A full conversion to NaNbO_3_ would probably have occurred given enough time, as others have succeeded in producing phase-pure NaNbO_3_ under similar conditions after a longer time.^26^ Despite many similarities between the reaction schemes in the two NaOH concentrations, a few differences are still observed; first, the stability of HNa_7_Nb_6_O_19_·15H_2_O is higher in the 9 M solution at all temperatures, which is consistent with the literature,^14^ as the transformation from T-Nb_2_O_5_ takes place earlier and the lifetime of the phase increases. Second, the opposite trend seems apparent for the following phases forming, resulting in a later onset of NaNbO_3_ formation in 12 M NaOH, especially at lower temperatures.

In all of the experiments, regardless of temperature and NaOH concentration, a transient phase (no. 3 in Figure 1) appears directly after the formation of HNa_7_Nb_6_O_19_·15H_2_O. This phase could not be matched with any structure file in the Inorganic Crystal Structure Database (ICSD), Crystallography Open Database (COD) or International Centre for Diffraction Data (ICDD), as specified in Table S1 in the Supporting Information. The first reflection (0.71 Å^–1^) of the HNa_7_Nb_6_O_19_·15H_2_O phase has the index (011) and seems to remain in this transient phase, while the two next major reflections (0.79 and 0.81 Å^–1^), indexed (101) and (110), disappear or shift. These three Bragg reflections originate from repeating [Nb_6_O_19_]^8–^ units, and the difference in the diffraction patterns might therefore indicate a change in how the units are tilted and separated by water and Na^+^ in certain directions. The following transformation of this transient phase leads to two new intermediate phases (nos. 4 and 5 in Figure 1) for NaOH concentrations of 9 and 12 M, respectively. These two phases could not be well matched with any structure in the ICSD, COD, or ICDD, as specified in Table S1 in the Supporting Information. As they both form from the same starting point and also evolve into the same structure in the next transformation (Na_2_Nb_2_O_6_·H_2_O), they most probably consist of the same building blocks. The diffraction patterns of the two phases have several similarities in the higher *Q*-range, but show a distinct difference at lower *Q*-values. The presence of the low-*Q* diffraction lines shows that these phases have large unit cells, which are typical for polyoxoniobate clusters. For the polyoxoniobate forming in 12 M NaOH, a line at a very low *Q* appears (0.63 Å^–1^), indicating the formation of larger clusters compared to the other intermediate phases. It is likely that the highly alkaline conditions might cause hydration of the [NbO_6_]^7–^ units, as [NbO_2_(OH)_4_]^3–^ is the dominating ion at high pH according to the literature.^46,47^ It is also known that high pH (>10) tends to destabilize or dissociate [Nb_6_O_19_]^8–^ units, leading to the potential assembly of other clusters from these dissociated fragments,^48,49^ such as heptaniobate [Nb_7_O_22_]^9–^, which can build quite large clusters.^50^ The indexing of these two polyoxoniobate phases both resulted in monoclinic crystal systems, as seen in Table S2 in the Supporting Information.

For temperatures below 285 °C for both NaOH concentrations, both of the polyoxoniobates forming in 9 and 12 M transform into Na_2_Nb_2_O_6_·H_2_O, previously observed under similar conditions.^13,16,20,22^ This phase can be described as staircase-like chains where each step is a [Nb_4_O_16_]^12–^ unit, and the chains are separated by water and Na^+^. For temperatures ≥285 °C, Na_2_Nb_2_O_6_·H_2_O is replaced by far less crystalline phases (nos. 7 and 8 in Figure 1). The diffraction patterns of these phases for the two NaOH concentrations are very similar and have similarities with both Na_2_Nb_2_O_6_·H_2_O and NaNbO_3_. Indexing of the version of the phase present in 9 M NaOH gave a triclinic crystal system, as seen from Table S2 in the Supporting Information. The broad reflection around 0.9 Å^–1^ (indexed (20-2) in Na_2_Nb_2_O_6_·H_2_O, representing the plane, which cuts through rows of neighboring chains) could originate from a partial collapse of the Na_2_Nb_2_O_6_·H_2_O unit cell, where the distance between chains becomes more disordered. This fits well with certain chains moving closer together as a result of water leaving the structure, which can be described as the formation of Na_2_Nb_2_O_6_·*x*H_2_O (*x* < 1). Diffraction patterns from the literature for a dehydrated version of Na_2_Nb_2_O_6_·H_2_O resembles the ones shown here.^12^ Additionally, it has been reported that Na_2_Nb_2_O_6_·H_2_O dehydrates and forms microporous Na_2_Nb_2_O_6_ as an intermediate phase before the formation of NaNbO_3_, at 282–290 °C,^12,35^ possibly explaining why this phase is observed in the temperature region ≥285 °C.^12^

**Figure 3:**
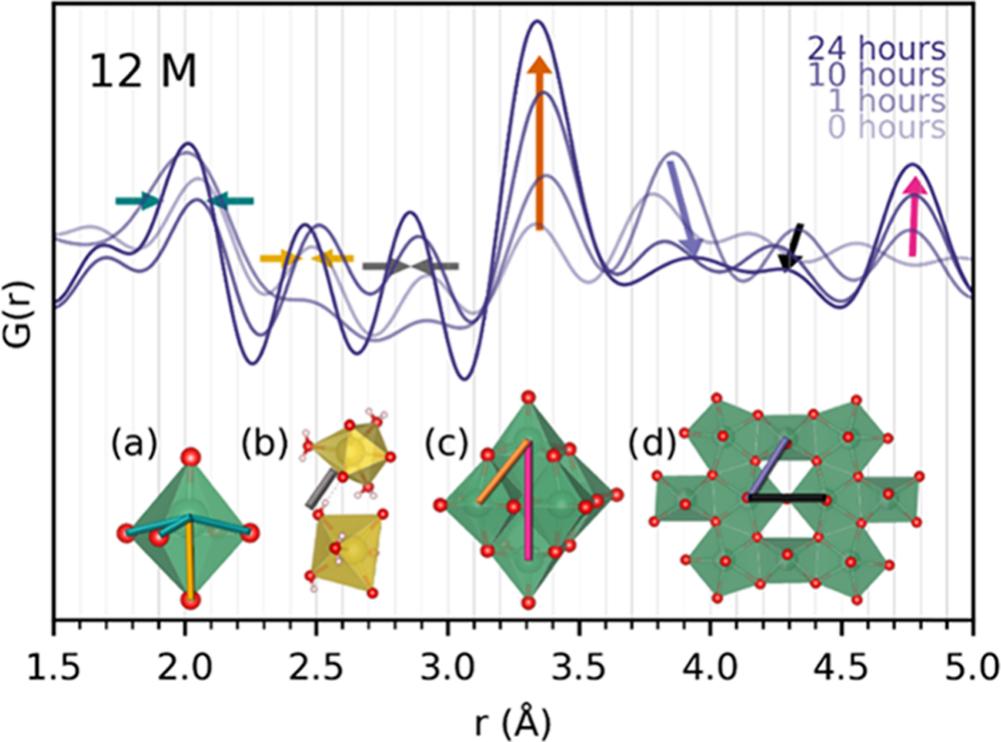
PDFs from total scattering data obtained for fresh and aged (1, 10, 24 h) suspensions with 12 M NaOH. Bond lengths and trends are indicated with similar colored lines and arrows for (a) a typical tetragonally distorted [NbO_6_]^7–^ octahedron, (b) neighboring Na–O units in the sodium hexaniobate structure, (c) a [Nb_6_O_19_]^8–^ Lindqvist ion, and (d) a fragment of the T-Nb_2_O_5_ structure. Two similarly colored arrows pointing toward one another indicate a narrowing of a peak, while one arrow pointing up- or downward indicates growth or shrinking of peak intensity.

To shed more light on the origin of the unassigned low-*Q* diffraction lines in the T-Nb_2_O_5_ precursor diffraction pattern in Figure 1 and illuminate their dependence on the time since the suspensions were prepared, *ex situ* total scattering data were obtained for unheated suspensions of T-Nb_2_O_5_ in 9 and 12 M NaOH aqueous solutions, aged for various times. Figure 3 presents the PDFs at a local range obtained from the total scattering data of fresh and aged (1, 10, 24 h) suspensions with 12 M NaOH. Longer *r*-range PDFs for suspensions with both 9 and 12 M NaOH are given in Figure S5a in the Supporting Information, along with the reduced structure functions, *F*(*Q*), in Figure S5b. From the bond lengths and trends included in Figure 3, we observe that the peak at approximately 2.0 Å, which represents the bond length between Nb and O in equatorial positions of octahedra or pentagonal bipyramids, is narrowing upon aging time. The peak at approximately 2.5 Å, originating from the long Nb–O bond in a tetragonally distorted octahedron, is increasing in intensity. Combined, these two observations indicate that the coordination of O around the Nb is becoming more defined with aging, likely forming more octahedra at the expense of pentagonal bipyramids. This is supported by the significant growth of peaks at 3.35 and 4.75 Å, coming from Nb–Nb distances of edge- and corner-sharing Nb–O octahedra. The two more subtle features at 3.8 and 4.2 Å, which correspond well with corner-sharing Nb–Nb distances involving at least one pentagonal bipyramid, seem to shift to higher *r*-values pointing to stretching of the bonds, before shrinking, further supporting the breaking up of pentagonal bipyramids. Such a breakup of the structure would require more oxygen entering the structure for the coordination around the Nb atoms to be maintained. This negative charge is likely to be neutralized by Na^+^ entering the structure, possibly explaining the increase of a peak at 2.85 Å, as this is the hydrogen-bond length expected between Na–O octahedra, presented by a gray line separating two Na–O octahedra in the inset (b) in Figure 3. When looking at the reduced structure functions, *F*(*Q*) in Figure S5b, the long-range order of the fresh suspensions matches well with the T-Nb_2_O_5_ structure, and there is a significant contribution from this structure even after 10 h of aging for both NaOH concentrations. Despite this, simultaneous fitting of the T-Nb_2_O_5_ and HNa_7_Nb_6_O_19_·15H_2_O to the PDFs obtained for 12 M NaOH suspensions (Figure S6 in the Supporting Information) shows that the 10 h aged suspension can be well described by the HNa_7_Nb_6_O_19_·15H_2_O structure alone. The PDFs for the 1 h aged 12 M suspension can be described well with the T-Nb_2_O_5_ structure, with only a small contribution from HNa_7_Nb_6_O_19_·15H_2_O, similar to the expected PDF of a Lindqvist ion. This suggests that the HNa_7_Nb_6_O_19_·15H_2_O structure is forming through a gradual change in the local crystal structure of T-Nb_2_O_5_, which would generate a large unit cell explaining the low-*Q* diffraction lines appearing for this phase in Figure 1.

**Figure 4:**
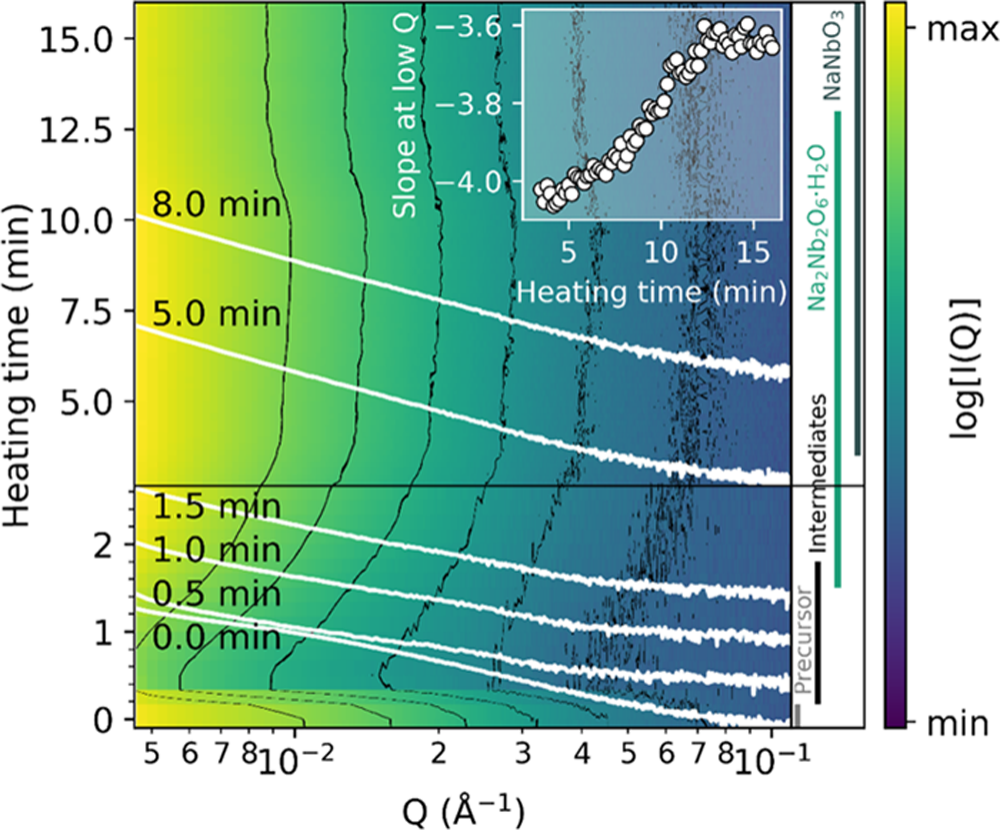
(a) *In situ* SAXS during the hydrothermal synthesis of NaNbO_3_ at 220 °C in 9 M NaOH solution as a function of heating time. The contours are accentuated with black lines, and a selection of the curves is drawn in white. The inset shows the slope of the curves in the low *Q*-range (0.0045–0.0055 Å^–1^). The phases specified in the right panel were determined by simultaneously recorded WAXS data as presented in Figure S7 in the Supporting Information.

To get a clearer view on how the different intermediate phases nucleate and grow into particles, simultaneous *in situ* SAXS/WAXS measurements were obtained. Figure 4 presents the *in situ* SAXS data for the hydrothermal synthesis of NaNbO_3_ at 220 °C in a 9 M NaOH solution. The wide-angle X-ray scattering (WAXS) data (presented in Figure S7 in the Supporting Information) were used to determine the phases present during the reaction, and these phases are specified in the right panel of Figure 4. The plot in the inset shows the slope of the curves in the low *Q*-range (0.0045–0.0055 Å^–1^), extracted by a fitting straight line to the double-logarithmic measured data in this low *Q*-range, for 4–16 min of heating. The SAXS data from the unheated precursor contains a broad distinct feature, which is assumed to originate from the T-Nb_2_O_5_ precursor particles or an amorphous phase. This feature disappears quickly upon heating and directly after its disappearance, a weak sign of another feature at approximately 0.01–0.03 Å^–1^ appears. This second feature is interpreted as the transient presence of a new set of particles, but the crystal structure(s) cannot be unequivocally identified from the WAXS data in Figure S7 due to the low time-resolution and limited *Q*-range. Even so, several reflections are appearing at similar *Q*-values (1.68, 2.05, 2.35, 2.40, 2.70, 2.80 Å^–1^) as for the two intermediate polyoxoniobates in Figure 1. The next phase identified with WAXS is Na_2_Nb_2_O_6_·H_2_O, and it can be seen that the SAXS slope at low *Q* increases steadily during the growth of this phase, before stabilizing at the same time (13 min) as the transformation from Na_2_Nb_2_O_6_·H_2_O to NaNbO_3_ completes.

**Figure 5:**
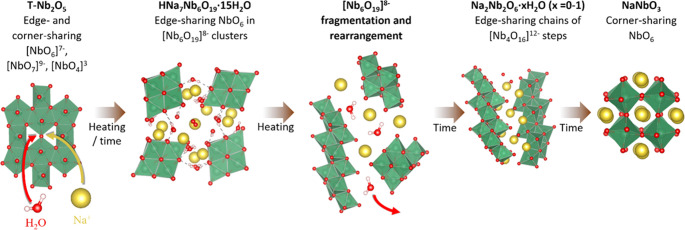
Proposed structural evolution during the hydrothermal synthesis of NaNbO_3_. Niobium in green, oxygen in red, sodium in yellow, and hydrogen in white.

To summarize, several intermediate phases are observed during the hydrothermal synthesis of NaNbO_3_. To understand the structural evolution from one phase to the next, one approach is to visualize the NbO*_x_* units of each phase, as seen in the proposed reaction scheme in Figure 5. The precursor T-Nb_2_O_5_ consists mostly of octahedra [NbO_6_]^7–^ and pentagonal bipyramids [NbO_7_]^9–^ with occasional tetrahedra [NbO_4_]^3–^. As the PDFs of the unheated T-Nb_2_O_5_ suspensions in Figure 3 shows, [Nb_6_O_19_]^8–^ units form at local scales rather quickly upon submerging the solid T-Nb_2_O_5_ in concentrated NaOH solutions, resulting in more [NbO_6_]^7–^ at the expense of [NbO_7_]^9–^ units, as soon as Na^+^ (along with charge-balancing oxygen atoms) and water enter the structure. Na^+^ and water could, for instance, enter the cavities of the T-Nb_2_O_5_ structure, resulting in the stretching and breaking of bonds between corner-sharing pentagonal bipyramids. This gradual change in the local environment is the foundation for forming HNa_7_Nb_6_O_19_·15H_2_O, with a Na/Nb ratio of ^7^/_6_. The Na/Nb ratio in Na_2_Nb_2_O_6_·H_2_O is 1, and thus the intermediate phases appearing between these two phases should have a ratio between 1 and ^7^/_6_, as a gradual expulsion could be expected. The water/Nb ratio should decrease successively from ^15^/_6_ in HNa_7_Nb_6_O_19_·15H_2_O to ^1^/_2_ in Na_2_Nb_2_O_6_·H_2_O, which could be the reason for the consistent shift to the right for the diffraction lines at low *Q*-values (except for the polyoxoniobate in 12 M NaOH). Previous literature predicts fragmentation and reorganization of [Nb_6_O_19_]^8–^ units to be the main event in highly alkaline solutions.^50^ Thus, a mechanism involving the building of the Na_2_Nb_2_O_6_·H_2_O staircase-like chains from [Nb_6_O_19_]^8–^ fragments is not unlikely. The other intermediate phases appearing between HNa_7_Nb_6_O_19_·15H_2_O and Na_2_Nb_2_O_6_·H_2_O could thus also consist of similar fragments. The transformation of Na_2_Nb_2_O_6_·*x*H_2_O (*x* < 1) to NaNbO_3_ expels the final water in the structure, causing the octahedra to become corner-sharing instead of edge-sharing for the charge to be distributed more evenly through the structure when water is not screening the charges any longer.

### Effects of Reaction Conditions on Crystallite Size and Unit Cell Volume

**Figure 6:**
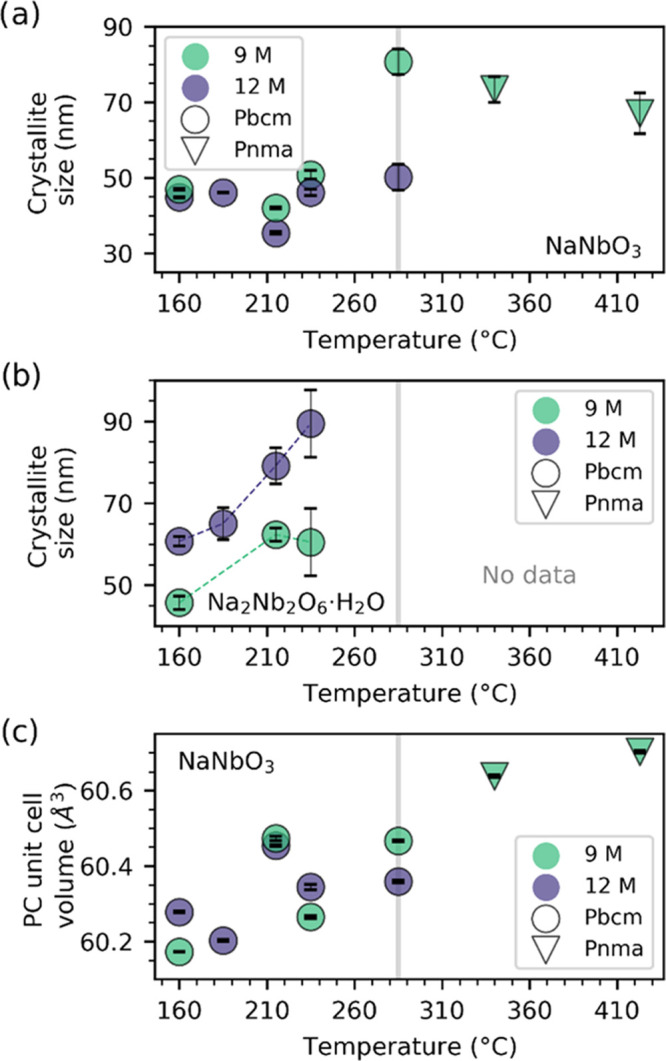
Crystallite size of (a) NaNbO_3_ and (b) Na_2_Nb_2_O_6_·H_2_O and (c) pseudo-cubic unit cell volume of NaNbO_3_ as a function of temperature prepared by hydrothermal synthesis in 9 and 12 M NaOH aqueous solutions. The gray line indicates the border between two regimes showing different behaviors. Marker types indicate different space groups of the NaNbO_3_ product.

Figure 6a shows the refined crystallite size of the final NaNbO_3_ product at various temperatures in 9 and 12 NaOH aqueous solutions. The refinements showed that the space group *Pbcm* gave a good fit for NaNbO_3_ formed below 340 °C, while for 340 °C and above, **Pnma** gave a better fit. This temperature is slightly lower than previously published bulk values for this phase transition, which predicts a transition upon heating around 370–400 °C.^37,51^ This suppression of the phase-transition temperature can be explained as a finite-size effect, often observed for ferroelectric oxides.^52^ Two different regimes are apparent for temperatures above and below ≈285 °C. Below this temperature, the crystallite size appears fairly temperature-independent with values of 35–50 nm, being slightly smaller for the experiments in 12 M NaOH solutions. Above ≈285 °C, the crystallite size is larger and seems to decrease with increasing temperature. The refined crystallite size of Na_2_Nb_2_O_6_·H_2_O in Figure 6b shows an increasing trend with increasing temperature and NaOH concentration, and the pseudo-cubic unit cell volume in Figure 6c increases with temperature and is affected by the NaOH concentration. The increase in the unit cell volume for higher temperatures is probably due to the elevated temperatures at which the measurements were performed. It is not likely to originate from a finite-size effect, as such an effect has previously shown to give an opposite trend for this materials class (*i*.*e*., smaller crystallites gives a larger unit cell).^52^

The difference in temperature effect on the crystallite size of NaNbO_3_ for reaction temperatures below and above ≈285 °C in Figure 6 shows that there is a difference in the growth mechanism for the two regimes. The features in the *in situ* SAXS signal in Figure 4 suggests that only one set of particles forms during the hydrothermal synthesis of NaNbO_3_ at 220 °C, as there is only one new feature to appear in the higher *Q*-range. This suggests that the particles formed in the beginning of the synthesis are converted directly into the next phases and not through a dissolution–precipitation mechanism at this temperature. The consistently larger crystallite size of Na_2_Nb_2_O_6_·H_2_O compared to NaNbO_3_ could thus be explained through a gradual conversion of the Na_2_Nb_2_O_6_·H_2_O particles to NaNbO_3_, by the expulsion of water from the structure.

### Effects of Reaction Conditions on the NaNbO_3_ Reaction Kinetics

**Figure 7:**
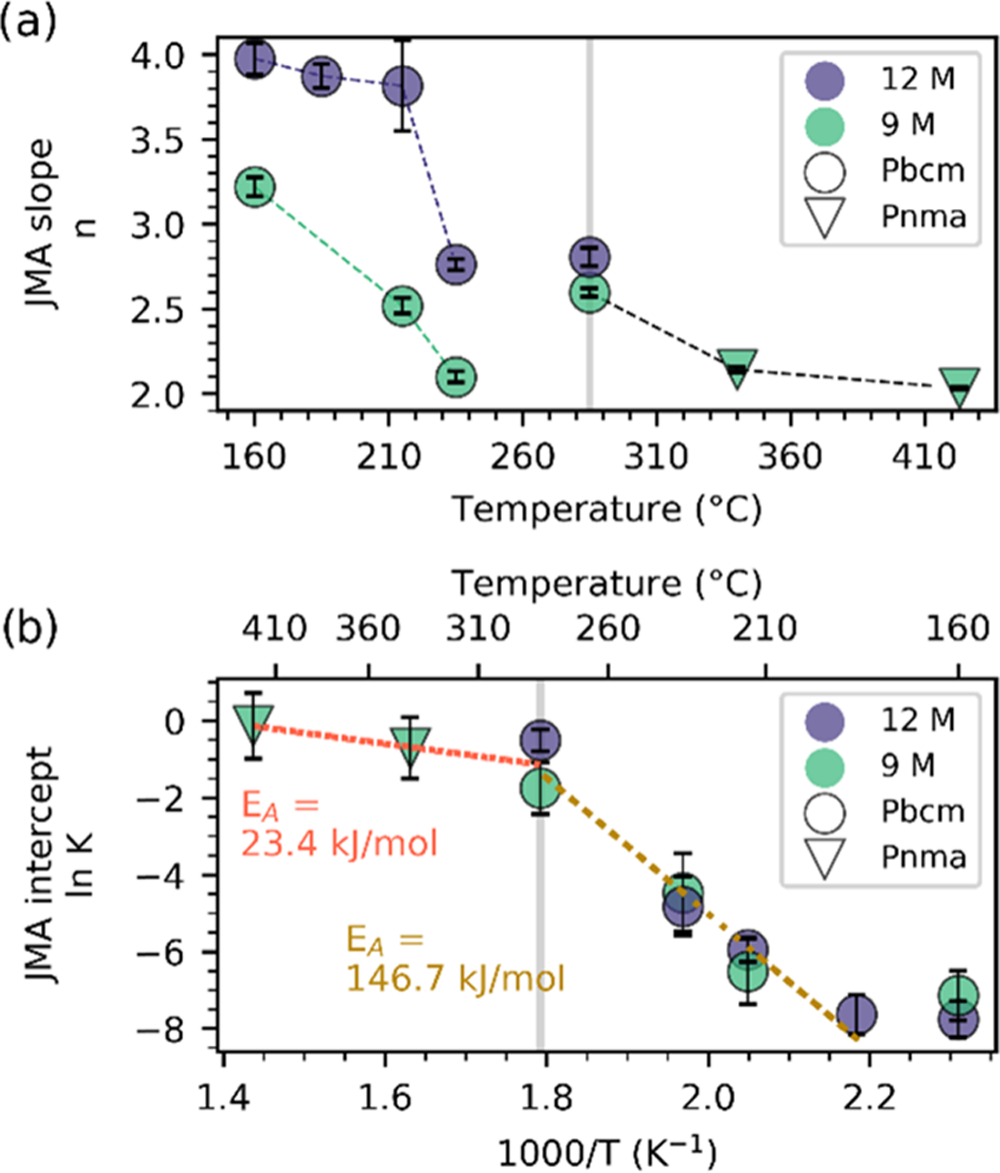
Kinetics of the final step in the reaction scheme for the hydrothermal synthesis of NaNbO_3_ at various temperatures in 9 and 12 M NaOH aqueous solutions represented by (a) the JMA slope and (b) the logarithm of the intercept K, shown as an Arrhenius plot. The gray line indicates the border between two regimes showing different behaviors. Marker types indicate different space groups of the NaNbO_3_ product.

The effects on the kinetics involved in the final step in the reaction scheme as well as the nucleation and growth of NaNbO_3_ are presented in Figure 7. The kinetics were quantified using the Johnson–Mehl–Avrami (JMA) equation α = 1 – *e*^–(*Kt*)^*n*^^, where α is the phase fraction, *K* is a rate constant, and *n* depends on the transformation mechanism.^39,40^ The JMA slope *n* and intercept *K* were calculated by transforming the phase fraction with the Sharp–Hancock method.^53^

The JMA slopes (*n*) in Figure 7a are in the range of 2–4, with a clear difference between the reactions at temperatures above and below ≈285 °C. Below ≈285 °C, the *n* values are strongly dependent on the NaOH concentration, having larger values for 12 M solutions. Such a difference in the *n* value could imply that there is a more restricted nucleation and/or growth of NaNbO3 in 9 M compared to 12 M suspensions.^53^ Above ≈285 °C, the difference between the two NaOH concentrations appears to decrease, although this should be confirmed with more experiments. The JMA intercepts (ln* K*) are presented in an Arrhenius plot in Figure 7b and seem to be independent of NaOH concentration. Again, a clear difference is seen between the two regimes below and above ≈285 °C, with significantly different Arrhenius slopes, from which the activation energy can be calculated. A significantly lower activation energy can be seen above ≈285 °C (23.4 kJ/mol), compared to below ≈285 °C (146.7 kJ/mol). This could be related to the less crystalline preexisting phase in the high-temperature region, giving a larger surface area and less rigid species for nucleation and growth.

By comparing the phase evolution in Figure 2 with the crystallite size in Figure 6, it is interesting that the three reactions resulting in the largest crystallite size were the reactions going through the poorly crystalline dehydrated Na_2_Nb_2_O_6_·*x*H_2_O, *x* < 1, phase on their way to NaNbO_3_. These reactions also have a lower activation energy and JMA slope compared to the reactions where the highly crystalline Na_2_Nb_2_O_6_·H_2_O phase is present. The low crystallinity of the dehydrated phase opens for a dissolution–precipitation-based transformation to NaNbO_3_, resulting in a decreasing crystallite size with decreasing temperature, which is what is observed.

The complex temperature-dependent reaction scheme and its effect on the kinetics and product crystallite size presented here underline the importance of *in situ* studies during the hydrothermal synthesis of oxides. The revealing of two distinctly different growth regimes may offer important insight when untangling the growth mechanisms, leading to the various sizes and morphologies resulting from the hydrothermal synthesis of NaNbO_3_.

## Conclusions

The entire reaction scheme, including several known and unknown intermediate phases, was observed during the hydrothermal synthesis of NaNbO_3_, and through observations over a large temperature range for two different NaOH concentrations, the relationship between them has been established. *Ex situ* PDF indicated that the T-Nb_2_O_5_ precursor partially transformed into HNa_7_Nb_6_O_19_·15H_2_O, before heating was initiated and a complete transition would occur quickly upon heating. After some time, depending on temperature and NaOH concentration, HNa_7_Nb_6_O_19_·15H_2_O, consisting of [Nb_6_O_19_]^8–^ clusters, was destabilized and fragmentized. These fragments are the most likely building blocks for the subsequent formation of polyoxoniobates, whose structure depended on the NaOH concentration. Following these polyoxoniobates was Na_2_Nb_2_O_6_·H_2_O, which appeared in a dehydrated form at temperatures ≥285 °C, before converting into the final phase, NaNbO_3_. The total reaction rate increased with decreasing NaOH concentration and increasing temperature, due to the increased stability of the intermediate polyoxoniobate phases. The final NaNbO_3_ particles had an orthorhombic structure with the *Pbcm* space group <340 °C, and **Pnma** ≥ 340 °C, showing suppression of the phase-transition temperature due to finite-size effects. Thermal expansion of the unit cell was observed, probably due to the elevated temperatures at which the measurements were performed.

Two distinctly different growth regimes for NaNbO_3_ were observed, based on the observed phase evolution and the resulting growth kinetics of NaNbO_3_, for temperatures below and above ≈285 °C. Below this temperature, the resulting crystallite size of NaNbO_3_ was independent of the reaction temperature and the NaOH concentration due to NaNbO_3_ growing at the expense of a highly crystalline intermediate phase, Na_2_Nb_2_O_6_·H_2_O. A high activation energy of 146.7 kJ/mol and pH- and temperature-dependent *n*-values were observed. When NaNbO_3_ grew at the expense of a less crystalline dehydrated intermediate phase for temperatures ≥285 °C, the resulting crystallite size was larger and showed a temperature-dependent trend typical for the dissolution–precipitation mechanism. The activation energy was significantly lower in this regime (23.4 kJ/mol) with *n*-values of ≈2.0–2.5.

## Supporting Information Available

VESTA visualizations of relevant structures; *in situ* heating profiles; phase evolution diagram with contour plots for the reactions in 12 M NaOH; contour plots of the two reactions at 160 °C with linear *y*-axis; pair-distribution functions and reduced structure functions from the *in situ* total scattering experiments; fits of niobate and hexaniobate structures to selected pair-distribution functions; and WAXS data recorded simultaneously with our SAXS data (PDF)
NaNbO_3__285C_12M (MP4)
