# Supplementary material for: Understanding the Hydrothermal Formation of NaNbO3: Its Full Reaction Scheme and Kinetics
Source: Inorg Chem. 2021 Mar 23;60(11):7632–40. doi: 10.1021/acs.inorgchem.0c02763 (PMC8188525; doi:10.1021/acs.inorgchem.0c02763)
Supplement: Supplementary file 1 — ic0c02763_si_001.pdf [file ic0c02763_si_001.pdf]

SUPPLEMENTARY INFORMATION:

Understanding the hydrothermal formation of $NaNbO_3$: Its full reaction scheme and kinetics

Susanne Linn Skjærvø,[a] Gary K. Ong,[b] Ola Gjønnes Grendal,[a] Kristin Høydalsvik Wells,[a] Wouter van Beek,[c] Koji Ohara,[d] Delia J. Milliron,[b] Satoshi Tominaka, [e] Tor Grande[a] and Mari-Ann Einarsrud[a,*]

a. Department of Materials Science and Engineering, NTNU Norwegian University of Science and Technology, 7491 Trondheim, Norway
b. McKetta Department of Chemical Engineering, The University of Texas at Austin, Texas 78712-1589, United States
c. Swiss-Norwegian Beamlines at the European Synchrotron Radiation Facility, 71 Avenue des Martyrs, 38043 Grenoble Cedex 9, France
d. Diffraction and Scattering Division, Center for Synchrotron Radiation Research, Japan Synchrotron Radiation Research Institute, 1-1-1 Kouto, Sayo-cho, Sayo-gun, Hyogo 679-5198, Japan
e. International Center for Materials Nanoarchitectonics (WPI-MANA), National Institute for Materials Science (NIMS), 1-1 Namiki, Tsukuba, Ibaraki 305-0044, Japan

*Corresponding author:

Mari-Ann Einarsrud

mari-ann.einarsrud@ntnu.no

phone: +47 48136521

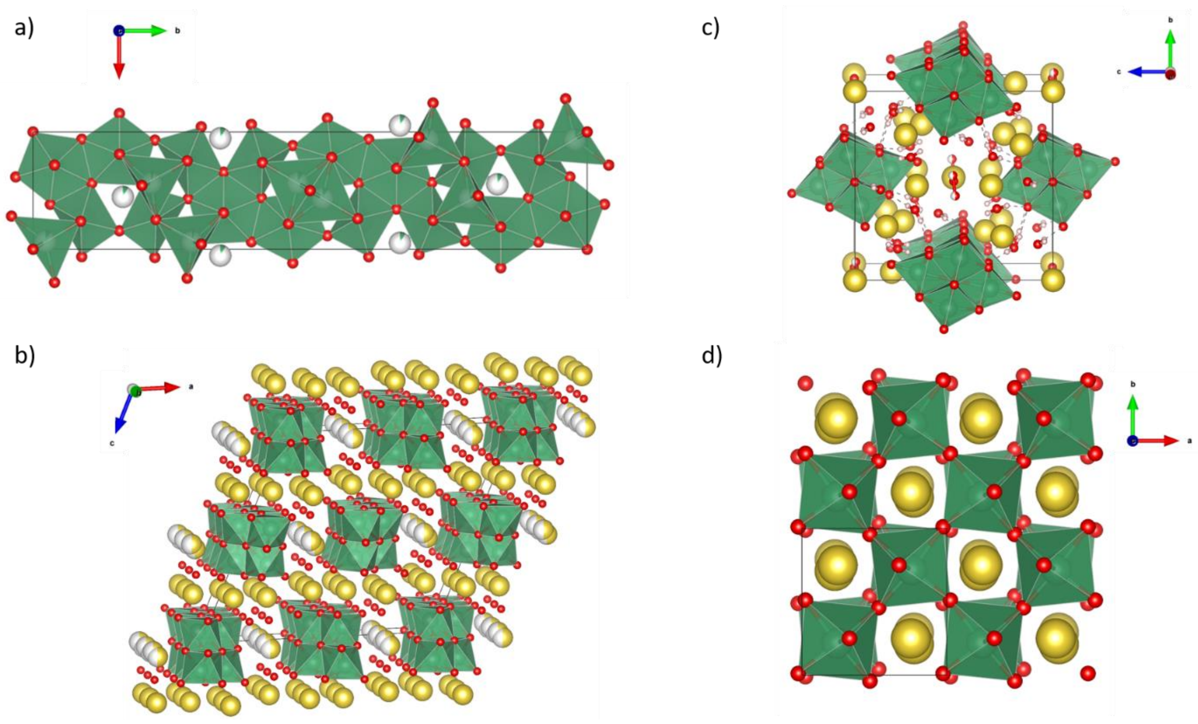

Figure S 1 Visualized crystal structures of the known phases during hydrothermal synthesis of $NaNbO_3$, drawn by the authors with VESTA.[1] The structures are a) T-Nb_2O_5,[2] b) $HNa_7Nb_6O_{19}\cdot 15H_2O$,[3] c) $Na_2Nb_2O_6\cdot H_2O$[4] and d) $NaNbO_3$,[5] where niobium, oxygen, sodium and hydrogen atoms are drawn with green, red, yellow and white.

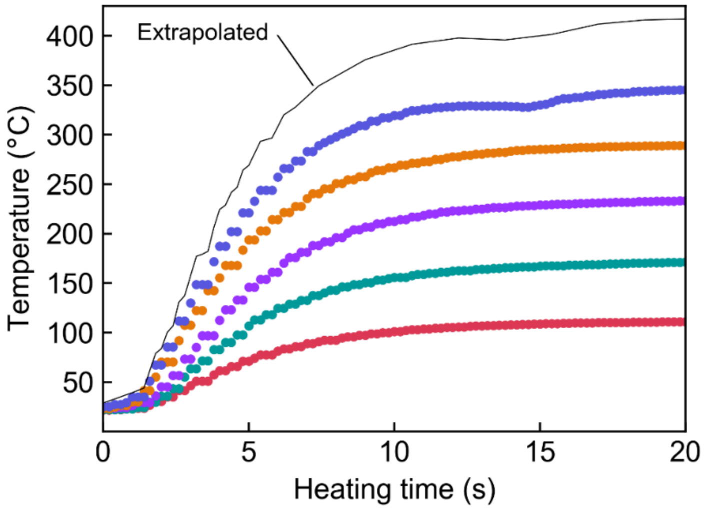

Figure S 2 Temperature profiles for different end temperatures, measured with a thermocouple inside a capillary filled with pure water and pressurized to 100 bar.

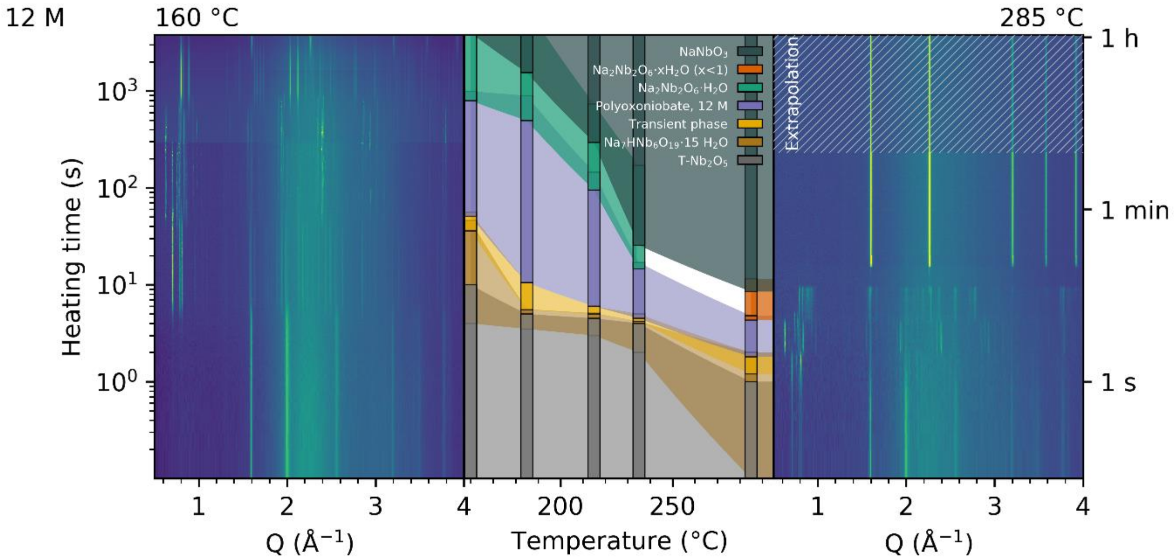

Figure S 3 Qualitative time-resolved phase evolution during hydrothermal synthesis of $NaNbO_3$ in 12 M NaOH aqueous solutions as a function of reaction temperature. Vertical bars represent real measured data, while the colored areas between them are logarithmic interpolations. The areas are transparent to make overlapping phases visible. The temporary loss of signal (at approx. 10-20 s of heating time) in the contour plot for 285 °C is due to movement of the sample inside the capillary, which can be seen from a video in the separate file “NaNbO3_285C_12M.mp4”, where heating is initiated after approximately 5 s.

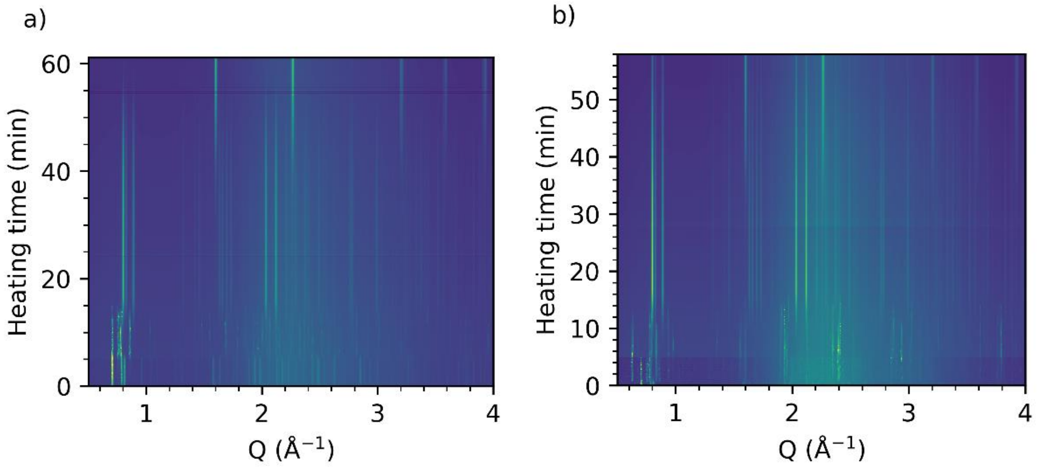

Figure S 4 Contour plots for reactions at 160 °C in a) 9 M and b) 12 M NaOH.

Table S 1 Structures found to be unsuccessful matches for the unidentified intermediate phases found during hydrothermal synthesis of $NaNbO_3$. The structures from the Inorganic Crystal Structure Database (ICSD)and Crystallography Open Database (COD) are all structures with 3-5 elements when searching for structures with the mandatory elements Nb or Ta together with O and the optional elements Na, K, H, F, W, V, Mo. The structures from the ICDD (International Centre for Diffraction Data) are all 4-element structures with Na/K, H, Nb and O.

#	Database	Code	Structure
**1**	ICSD	26620	(K2 (Nb F5 O)) (K H F2)
**2**	ICSD	434604	Na8 (Nb6 O19) (H2 O)25
**3**	ICSD	30722	Na7 Nb15 W13 O80
**4**	ICSD	200967	Na7 (H3 O) (Nb6 O19) (H2 O)14
**5**	ICSD	417857	Na7 (H Nb6 O19) (H2 O)15
**6**	ICSD	24819	Na5 Nb O5
**7**	ICSD	72298	Na5 (Nb O5)
**8**	ICSD	202574	Na3 Nb12 O31 F
**9**	ICSD	62378	Na3 Nb12 O31 F
**10**	ICSD	6116	Na3 Nb O4
**11**	ICSD	67513	Na3 K3 (Nb8 P5 O35)
**12**	ICSD	24399	Na3 H F2 Nb F5 O2
**13**	ICSD	35761	Na3 (Nb F6 O)
**14**	ICSD	24404	Na2 Nb F5 O2 (H2 O)2
**15**	ICSD	166951	Na2 (Nb4 O11)
**16**	ICSD	18305	Na2 (Nb4 O11)
**17**	ICSD	183855	Na2 (Nb4 O11)
**18**	ICSD	55415	Na2 (Nb2 O6) (H2 O)
**19**	ICSD	23333	Na2 (Nb F5 O2) (H2 O)
**20**	ICSD	48165	Na2 (Nb F5 O)
**21**	ICSD	24882	Na13 Nb35 O94
**22**	ICSD	38004	Na0.35 K0.65 Nb O3
**23**	ICSD	48105	Na Nb7 O18
**24**	ICSD	61364	Na Nb3 O5 F
**25**	ICSD	24300	Na Nb13 O33
**26**	ICSD	85052	Na Nb10 O18
**27**	ICSD	24109	Na (Nb6 O15 F)
**28**	ICSD	24108	Na (Nb6 O15 (O H))
**29**	ICSD	202400	Na (Nb3 O8)
**30**	ICSD	193826	Na (Nb2 O5 (O H)) (H2 O)3
**31**	ICSD	45813	Na (Nb W O6) (H2 O)0.5
**32**	ICSD	45802	Na (Nb W O6)
**33**	ICSD	73111	Na (Nb O2)
**34**	ICSD	23238	Na (Nb F2 O2)
**35**	ICSD	391371	K8 (Nb6 O19) (H2 O)16
**36**	ICSD	89492	K8 (H4 Nb4 W8 O36 (O2)4) (H2 O)18
**37**	ICSD	391372	K7 (H Nb6 O19) (H2 O)10
**38**	ICSD	409464	K6 Nb10.8 O30
**39**	ICSD	83600	K6 (Nb10.88 O30)
**40**	ICSD	73834	K5.34 Mo9.22 Nb0.77 O30
**41**	ICSD	434606	K5 Na3 (Nb6 O19) (H2 O)9
**42**	ICSD	20651	K5 (Nb3 O3 F14) (H2 O)

**43**	ICSD	201017	K5 (Nb O3)4 F
**44**	ICSD	35030	K4 Nb6 O17
**45**	ICSD	201721	K3 Nb7 O19
**46**	ICSD	62550	K3 Nb7 O19
**47**	ICSD	2298	K3 (Nb8 O21)
**48**	ICSD	72514	K3 (Nb8 O21)
**49**	ICSD	82188	K3 (Nb6 V O19)
**50**	ICSD	26634	K3 (Nb O F6)
**51**	ICSD	30405	K3 (Nb (O2)4)
**52**	ICSD	54960	K2.6 Nb11.6 O30
**53**	ICSD	193496	K2.05 (Nb3.74 O9.81)
**54**	ICSD	62130	K2 Nb10 W7 O47
**55**	ICSD	183827	K2 Na Nb O2 F4
**56**	ICSD	25748	K2 Na Nb O2 F4
**57**	ICSD	71162	K2 (Nb O2 F5) (H2 O)
**58**	ICSD	420848	K13 Na3 H Nb27 O78 (H2 O)25
**59**	ICSD	87532	K1.68 Nb4 O3 (O2 F) F
**60**	ICSD	261134	K1.42 H1.66 Nb2 O6 (O H)0.58 (H2 O)0.42
**61**	ICSD	87531	K1.25 Nb4 O3 (O2 F) F
**62**	ICSD	157872	K0.94 H0.06 (Nb O3)
**63**	ICSD	157871	K0.91 H0.09 (Nb O3)
**64**	ICSD	261139	K0.88 H1.66 Nb2 O6 (O H)0.54 (H2 O)1.12
**65**	ICSD	261137	K0.88 H1.66 Nb2 O6 (O H)0.54 (H2 O)1.04
**66**	ICSD	261140	K0.88 H1.66 Nb2 O6 (O H)0.54 (H2 O)1.04
**67**	ICSD	162410	K0.43 ((Nb0.106 W0.894) O3)
**68**	ICSD	71218	K Nb8 O14
**69**	ICSD	31994	K Nb3 O8
**70**	ICSD	421274	K Nb2 O5 F (H2 O)1.2
**71**	ICSD	403491	K Nb W2 O9
**72**	ICSD	18065	K Nb (W O6) (H2 O)
**73**	ICSD	195786	K Nb (V2 O8)
**74**	ICSD	422708	K Na Nb O F5
**75**	ICSD	241671	K Na (Nb O F5)
**76**	ICSD	423273	K Na (Nb O F5)
**77**	ICSD	423274	K Na (Nb O F5)
**78**	ICSD	170693	K (Nb5 O13)
**79**	ICSD	246141	K (Nb W2 O9)
**80**	ICSD	96975	K (Nb W O6) (H2 O)
**81**	ICSD	423165	(K3 (Ta O4)) (K3 (Ta F4 O2))
**82**	ICSD	32627	H2 (Ta2 O6)
**83**	ICSD	246142	K (Ta W2 O9)
**84**	ICSD	9636	K (Ta2 O5 F) (H2 O)
**85**	ICSD	2462	K (Ta5 O13)
**86**	ICSD	421278	K Ta2 O5 (O H)
**87**	ICSD	188983	K1.67 (Ta2 O5.54)
**88**	ICSD	188984	K1.93 (Ta2 O5.90)
**89**	ICSD	425506	K2 (Ta4 O11)
**90**	ICSD	50515	K2 Ta15 O32
**91**	ICSD	15866	K2 Ta2 O3 F6
**92**	ICSD	8204	K2 Ta4 F4 O9

**93**	ICSD	73104	K3 (Ta (O2)4)
**94**	ICSD	403172	K3 Ta O2 F4
**95**	ICSD	2209	K6 Ta10.8 O30
**96**	ICSD	14013	K6 Ta3 (O2)3 O F13 (H2 O)
**97**	ICSD	250119	K6 Ta6.27 O15 F7.4
**98**	ICSD	100188	K6 Ta6.5 O14.5 F9.5
**99**	ICSD	93934	K6 Ta6.5 O15 F7.56
**100**	ICSD	250121	K6 Ta6.88 O15 F8.2
**101**	ICSD	250120	K6 Ta7.05 O15 F7.1
**102**	ICSD	391056	K7 Na (Ta6 O19) (H2 O)14
**103**	ICSD	391057	K8 (Ta6 O19) (H2 O)16
**104**	ICSD	56877	K8 (Ta6 O19) (H2 O)16
**105**	ICSD	45286	K8 Ta6 O19 (H2 O)16
**106**	ICSD	247163	Mo2 Ta2 O11
**107**	ICSD	45814	Na (Ta W O6) (H2 O)0.5
**108**	ICSD	45809	Na (Ta W O6) (H2 O)
**109**	ICSD	63204	Na Ta3 O8
**110**	ICSD	410260	Na0.74 (Ta3 O6)
**111**	ICSD	4080	Na2 Ta2 O5 F2
**112**	ICSD	201714	Na2 Ta4 O11
**113**	ICSD	40984	Na2 Ta4 O11
**114**	ICSD	431820	Na3 (Ta O8) (H2 O)14
**115**	ICSD	789	Na3 Ta O4
**116**	ICSD	72297	Na5 (Ta O5)
**117**	ICSD	417856	Na8 (Ta6 O19) (H2 O)15
**118**	ICSD	422177	Na8 (Ta6 O19) (H2 O)24.5
**119**	ICSD	421840	Na8 (Ta6 O19) (H2 O)26
**120**	ICSD	421841	Na8 (Ta6 O19) (H2 O)26
**121**	COD	2020375	Na7 Nb15 W13 O80
**122**	COD	1534392	Na5 Nb O5
**123**	COD	1530277	Na3 Nb12 O31 F
**124**	COD	1528601	Na2 Nb4 O11
**125**	COD	7212163	Na2 Nb4 O11
**126**	COD	1539126	Na2 Nb F5 O2 (H2 O)2
**127**	COD	1534376	Na13 Nb35 O94
**128**	COD	5910192	Na Nb6 O15 F
**129**	COD	9012849	Na Nb3 O8
**130**	COD	5910220	Na Nb13 O33
**131**	COD	1001745	Na (Nb3 O8)
**132**	COD	1011064	Na (Nb O3)
**133**	COD	1542166	Na (Nb O2)
**134**	COD	1538463	K2 Na Nb O2 F4
**135**	COD	4110148	K Na Nb O F5
**136**	COD	4118533	H97 K Na2 Nb24 O110
**137**	COD	4118534	H188 K2 Na2 Nb32 O176
**138**	COD	7005979	2(H Nb6 O19) (14 Na) (30 H2O)
**139**	COD	1001030	Na2 Ta2 O6
**140**	COD	1001166	H2 (H2 O) Ta2 O6
**141**	COD	1518102	Na2 O11 Ta4
**142**	COD	1535074	K7 Na (Ta6 O19) (H2 O)14

**143**	ICDD	04-016-8512	H0.83K0.44NbO3(OH)0.27(H2O)0.57
**144**	ICDD	01-076-7830	NbO1.95F1.05
**145**	ICDD	01-075-2947	Nb12O29
**146**	ICDD	00-043-1173	NaNbO2
**147**	ICDD	00-034-1492	NaNb7O18
**148**	ICDD	00-019-1223	NaNb6O15OH
**149**	ICDD	00-019-1222	NaNb6O15F
**150**	ICDD	04-021-1085	NaNb2O5(OH)(H2O)3
**151**	ICDD	00-020-1145	Na2Nb4O11
**152**	ICDD	04-014-2939	Na2Nb2O6(H2O)
**153**	ICDD	04-012-6659	KNb3O8
**154**	ICDD	01-080-1921	KNb3.76O6
**155**	ICDD	01-082-3987	KNa(NbOF5)
**156**	ICDD	04-018-1316	K8Nb6O19(H2O)16
**157**	ICDD	04-018-1317	K7Nb6O18(OH)(H2O)10
**158**	ICDD	04-010-9048	K5Nb14.6O39(H2O)5
**159**	ICDD	04-010-1844	K3Nb8O21
**160**	ICDD	00-038-1499	K3Nb7O19
**161**	ICDD	04-011-4797	K3Nb5.45O15
**162**	ICDD	01-080-6278	K2NaNbO2F4
**163**	ICDD	01-073-7471	K2.6Nb11.6O30
**164**	ICDD	01-079-8169	K0.88H1.66Nb2O6(OH)0.54(H2O)1.12
**165**	ICDD	04-021-4698	HK7Nb6O19(H2O)13

Table S 2 Indexing of the unidentified polyoxometalate phases in 9 and 12 M suspensions, and the assumed dehydrated phase ($Na_2Nb_2O_6 \cdot xH_2O$ ($x < 1$)). The results were obtained using McMaille[6] for the 20 most intense diffraction lines.

Phase name	Crystal system	Lattice parameters (Å)		Space group used in refinement	R_{Bragg} (%)
**Polyoxometalate (9 M)**	Monoclinic	a = 16.7078(4)<br>b = 15.1086(4)<br>c = 11.8281(3)	β = 119.591(2)	P121	13.6
**Polyoxometalate (12 M)**	Monoclinic	a = 10.2031(4)<br>b = 17.0235(4)<br>c = 16.4707(4)	β = 100.477(3)	P121	20
**Dehydrated phase ($Na_2Nb_2O_6 \cdot xH_2O$ ($x < 1$))**	Triclinic	a = 9.6379(6)<br>b = 8.9727(4)<br>c = 8.8142(4)	α = 65.867(3)<br>β = 62.428(3)<br>γ = 75.305(4)	P1	7

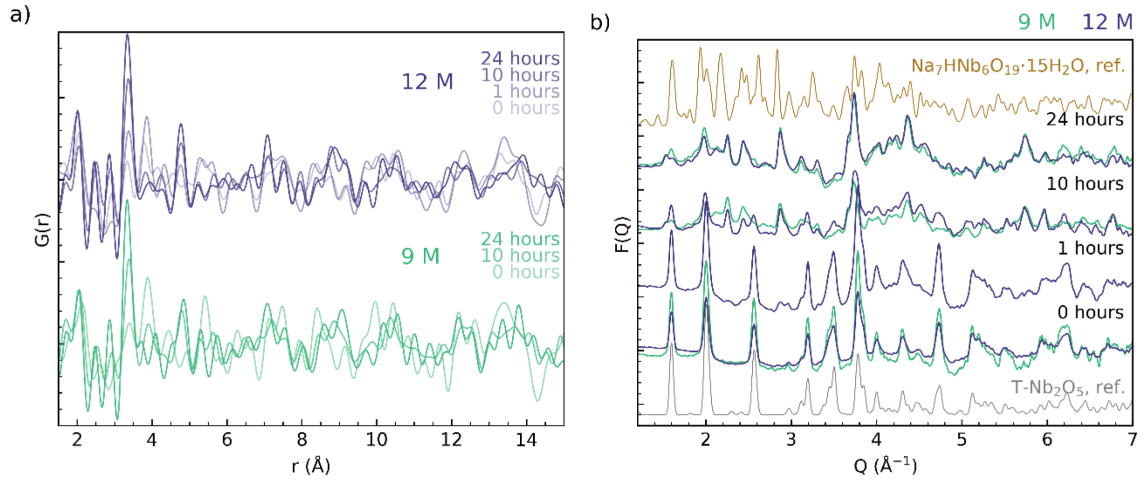

Figure S 5 a) Pair-distribution functions, *G(r)* and b) reduced structure functions, *F(Q)* from the recorded total scattering data for fresh and 1, 10, 24 h aged suspensions of T-Nb_2O_5 in 9 and 12 M NaOH solutions at ambient temperature and pressure.

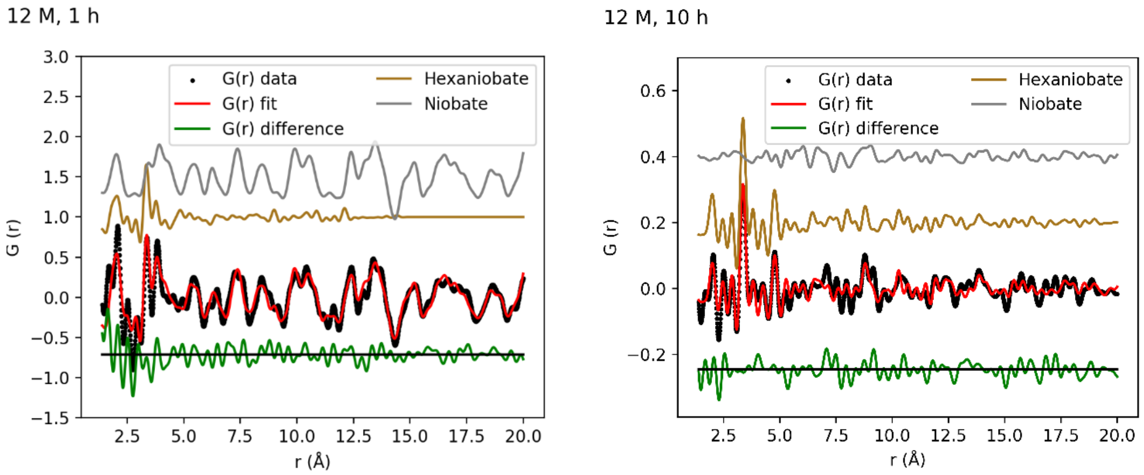

Figure S 6 Two-phase fitting for hexaniobate ($HNa_7Nb_6O_{19}\cdot15H_2O$) [3] and niobate (T-$Nb_2O_5$) [2] to the obtained pair-distribution functions (PDF) for 1 and 10 h aged suspensions with 12 M NaOH. The 1 h aged suspension is described well by the niobate structure, with only a small contribution from hexaniobate, similar to the expected PDF of a Lindqvist ion. For the 10 h aged suspension, the PDF can be described well with only the hexaniobate structure. The refined parameters for both structures were scale factor, unit cell parameter, delta2, crystallite size and isotropic displacement parameters.

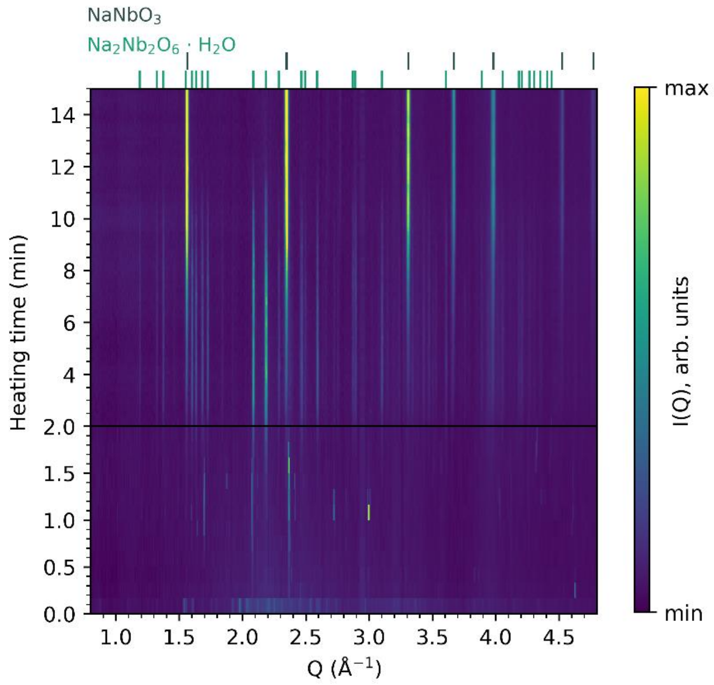

Figure S 7 In situ PXRD during the hydrothermal synthesis of $NaNbO_3$ at 220 °C in 9 M NaOH solution as a function of heating time recorded simultaneously with the *in situ* SAXS signal. The reference patterns for $Na_2Nb_2O_6{\cdot}H_2O$ [4] and $NaNbO_3$ [5] are shown. The strong transient peaks appearing after 1-2 min of heating could not be identified due to insufficient statistics.

References

(1) Momma, K.; Izumi, F. VESTA 3 for Three-Dimensional Visualization of Crystal, Volumetric and Morphology Data. *J. Appl. Cryst.* **2011**, *44* (6), 1272–1276. https://doi.org/10.1107/S0021889811038970.

(2) Kato, K.; Tamura, S. Die Kristallostruktur von T-Nb_2O_5. *Acta Crystallogr.* **1975**, *B 31* (3), 673–677. https://doi.org/10.1107/S0567740875003603.

(3) Goiffon, A.; Philippot, E.; Maurin, M. Structure Cristalline Du Niobate 7/6 de Sodium $(Na_7)(H_3O)Nb_6O_{19}{\cdot}14\,H_2O$. *Rev. Chim. Minérale* **1980**, *17*, 466–476.

(4) Xu, H.; Nyman, M.; Nenoff, T. M.; Navrotsky, A. Prototype Sandia Octahedral Molecular Sieve (SOMS) $Na_2Nb_2O_6{\cdot}H_2O$: Synthesis , Structure and Thermodynamic Stability. *Chem. Mater.* **2004**, *16*, 2034–2040.

(5) Mishra, S. K.; Choudhury, N.; Chaplot, S. L.; Krishna, P. S. R.; Mittal, R. Competing Antiferroelectric and Ferroelectric Interactions in $NaNbO_3$: Neutron Diffraction and Theoretical Studies. *Phys. Rev. B* **2007**, *76* (024110), 1–8. https://doi.org/10.1103/PhysRevB.76.024110.

(6) Le Bail, A. Monte Carlo Indexing with McMaille. *Powder Diffr.* **2004**, *19* (03), 249–254. https://doi.org/10.1154/1.1763152.
